# Centrality of Early Synaptopathy in Parkinson’s Disease

**DOI:** 10.3389/fneur.2018.00103

**Published:** 2018-03-01

**Authors:** Paola Imbriani, Tommaso Schirinzi, Maria Meringolo, Nicola B. Mercuri, Antonio Pisani

**Affiliations:** ^1^Department of Systems Medicine, University of Rome “Tor Vergata”, Rome, Italy; ^2^Laboratory of Neurophysiology and Plasticity, Fondazione Santa Lucia (IRCCS), Rome, Italy

**Keywords:** Parkinson’s disease, cellular mechanisms, synaptopathy, dopamine transmission, animal models

## Abstract

Significant advances have been made in the understanding of the numerous mechanisms involved in Parkinson’s disease (PD) pathogenesis. The identification of PD pathogenic mutations and the use of different animal models have contributed to better elucidate the processes underlying the disease. Here, we report a brief survey of some relevant cellular mechanisms, including autophagic–lysosomal dysfunction, endoplasmic reticulum stress, and mitochondrial impairment, with the main aim to focus on their potential convergent roles in determining early alterations at the synaptic level, mainly consisting in a decrease in dopamine release at nigrostriatal terminals and loss of synaptic plasticity at corticostriatal synapses. In a number of experimental models, this synaptopathy has been shown to be an initial, central event in PD pathogenesis, preceding neuronal damage, thereby representing a valuable tool for testing potential disease-modifying treatments.

## Introduction

Parkinson’s disease (PD) is a common neurodegenerative disorder, currently affecting 1% of the population above 60 years (1) and characterized by progressive motor deficits, including akinesia (or bradykinesia), rigidity, resting tremor, and postural instability (2). The neuropathological hallmarks of PD are the progressive loss of substantia nigra pars compacta (SNpc) dopaminergic neurons (DAns) and the presence of intraneuronal α-synuclein cytoplasmic inclusions, termed Lewy bodies (3). In the past two decades, a number of pathogenic mutations associated with PD have been identified (4), improving our understanding of pathogenic disease mechanisms. Many PD-related genes, such as *SNCA, PINK1, GBA1*, have a crucial role in different cellular mechanisms that have proven to be involved in PD, including autophagy/lysosome pathway, endoplasmic reticulum (ER) stress, and mitochondrial impairment (5). In this regard, the purpose of this review is to provide a brief overview of the most relevant pathogenic mechanisms, but with a specific focus on clues supporting early synaptic dysfunction as a functional and structural event that could represent a final convergent phenomenon for multiple distinct processes. Comprehensive review of PD-related pathogenic mechanisms is beyond the scope of this survey, and we refer the readers to other recent excellent reviews (5, 6).

## Synaptopathy in PD

**Figure 1:**
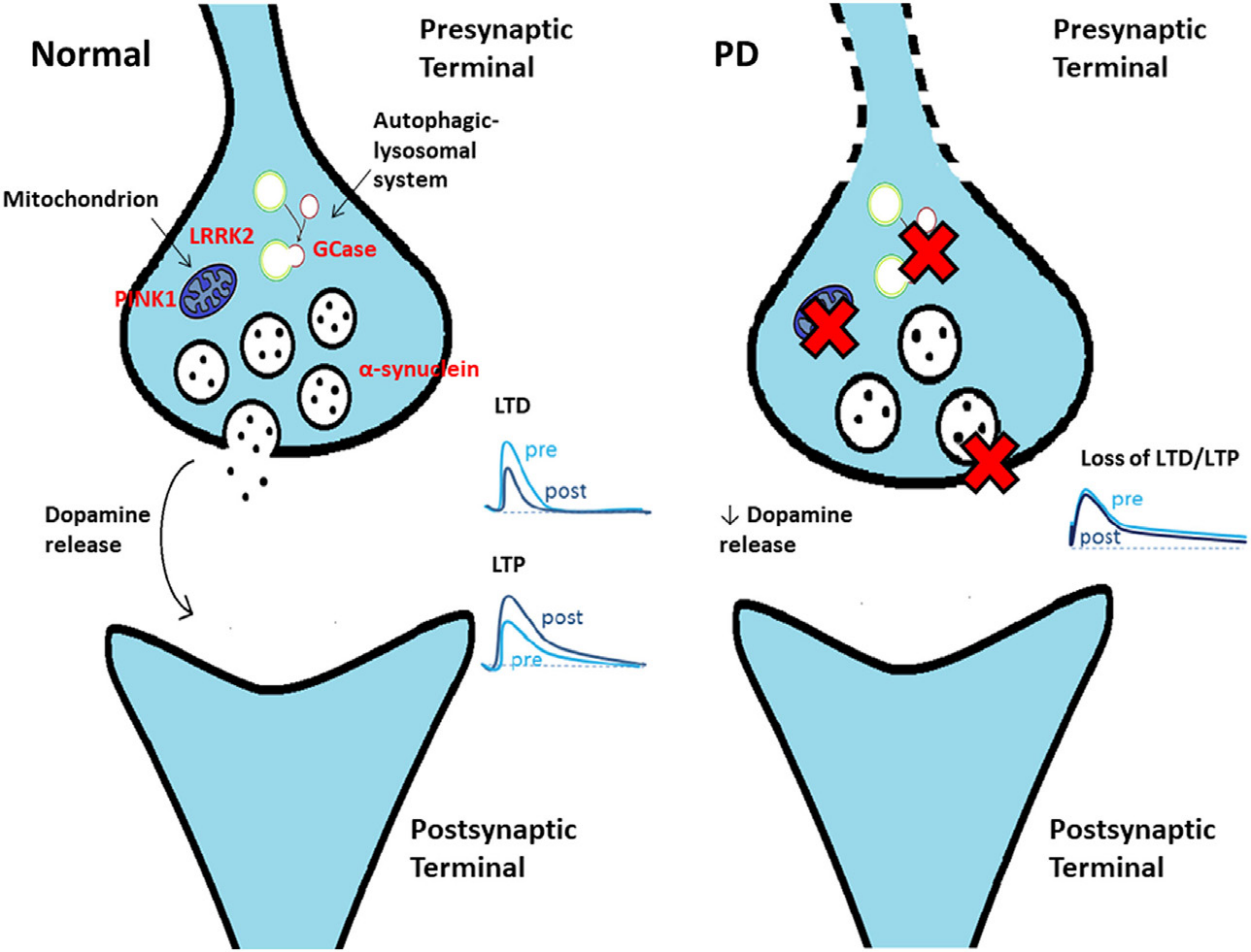
Schematic cartoon showing a synaptic terminal in control conditions (left) and in Parkinson’s disease (PD) (right). Several mechanisms contribute to early dysfunction at the terminal level, causing an impaired dopamine release. As a consequence, synaptic plasticity at corticostriatal synapses [long-term depression (LTD) and long-term potentiation (LTP)] is impaired in PD.

Despite considerable progress in our understanding of the aberrant mechanisms involved in PD pathogenesis, some key questions remain unanswered. Among these, a central issue is to establish the precise sequence of events at the cellular level and where the pathogenic process begins. Multiple lines of evidence suggest that the primary site of α-synucleinopathy is represented by the synaptic terminal, with the occurrence of an early synaptic impairment that precedes axon degeneration and with subsequent retrograde progression through a dying-back mechanism (7) (Figure 1). This “synaptopathy” described at the cellular level could correspond to an early, presymptomatic time window in patients, when only a 30–50% decrease in striatal dopamine levels can be detected, providing evidence for a powerful ability of the motor system to compensate (8). The concept of synaptopathy is closely related to α-synuclein, the major constituent of Lewy body, which, in physiological conditions, is primarily localized to the presynaptic terminals, where it affects the fusion and clustering of synaptic vesicles, thus influencing neurotransmitter release (9–11). Evidence on how α-synuclein plays a crucial role in synaptic function and plasticity comes from several studies on animal models. In a transgenic mouse model of α-synucleinopathy (αSyn 1-120 mice), α-synuclein aggregates were detected at striatal dopaminergic terminals, with an impairment of dopamine release from nigrostriatal synaptic terminals, even in the absence of nigral DAn loss (12). In another model, a bacterial artificial chromosome (BAC) transgenic mouse with overexpression of human wild-type α-synuclein (SNCA-OVX), a clear time-dependent progression was observed: in 3-month-old mice, in spite of the absence of overt neuropathology, early deficits in dopamine release in the dorsal striatum and increased clustering of vesicles in dopamine terminals were found (13). Conversely, at 18 months, mice showed motor deficits, loss of dopamine neurons, and a reduced firing rate in the remaining SNpc dopamine neurons, further indicating synaptic dysfunction as an early event. Of relevance, this feature does not apply only to α-synuclein models. Many other PD animal models, including those based on the administration of the “classical” neurotoxins 6-OHDA and MPTP, and the ones with genetic mutations not involving α-synuclein, have contributed to identify synaptic dysfunctions occurring at early stages of the disease. In a 6-OHDA model of early PD with partial denervation and mild motor alterations, the decreased level of dopamine observed was responsible for a selective impairment of corticostriatal synaptic plasticity recorded from spiny projection neurons (SPNs), with a specific deficit of long-term potentiation (LTP) and with sparing of long-term depression (LTD) (14). Similar corticostriatal synaptic plasticity impairments were also found by Chou et al. (15), who performed electrophysiological recordings from SPNs and from SNpc dopaminergic cells of 8–9-month-old *LRRK2* (G2019S mutation) transgenic mice. *LRRK2* is a multidomain protein with kinase activity, whose mutations are involved in autosomal dominant forms of PD (16). The function of LRRK2 has not been fully elucidated, although strong evidence implicates a role in intracellular trafficking, vesicular recycling, and modulation of synaptic transmission (17). In line with this, Chou et al. identified an early decrease in spontaneous firing frequency of SNpc dopaminergic cells, without gross degeneration of nigrostriatal terminals, and impaired evoked dopamine release, with subsequent deficit in LTD induction in striatal neurons (15). Moreover, a recent study on *LRRK2* BAC transgenic rats revealed alterations to dopamine circuit function, in the form of L-DOPA-responsive motor dysfunction, a reduction in SNpc dopamine neurons burst firing, and an impaired striatal dopamine release, occurring in the absence of neurodegeneration or abnormal protein accumulation (18). Besides the examples reported so far, the concept of synaptopathy can be extended to various PD models and appears to be linked to different cellular mechanisms, as discussed in the following sections.

## Dysfunctional Autophagy–Lysosome System and the Role of *GBA1* Mutations

Autophagy, acting through lysosomal degradation, represents the main proteolytic system in neurons (19). An abnormal autophagic activity leads to the accumulation of aberrant proteins and toxic components (20), contributing to the neurodegeneration observed in several diseases, including PD (21). In PD, dysfunctional autophagy is responsible for the accumulation of α-synuclein: specifically, two types of autophagy (macroautophagy and chaperone-mediated autophagy), both involved in α-synuclein degradation, appear to be impaired in PD (22). In addition, growing evidence indicates that excessive α-synuclein itself blocks these degradation pathways, promoting α-synuclein aggregation (23). Experimental findings show that Lewy body-like aggregates are able to resist to macroautophagy degradation by impairing clearance of autophagosomes (24) and that two α-synuclein familial mutations, *SNCA-A30P* and *SNCA-A53T*, can alter the chaperone-mediated autophagy pathway (25). In the recent past, a growing body of evidence suggests a prominent role for decreased glucocerebrosidase (GCase) activity in autophagic failure and subsequent α-synuclein accumulation in PD (26). GCase is a lysosomal enzyme encoded by the *GBA1* gene, ubiquitously expressed in the brain with some regional variations (27), whose homozygous or heterozygous compound mutations cause Gaucher disease (GD), with the accumulation of glycolipid substrate (28). Of note, heterozygous *GBA1* mutations represent the most relevant risk factor for PD, since they can be found in approximately 5–10% of idiopathic PD patients (29). The majority of *GBA1* mutations are associated with reduced GCase levels (30), with milder mutations responsible for slightly diminished enzyme levels, conferring a much lower risk of PD than mutations causing severe enzymatic dysfunction (31). The molecular mechanisms underlying the increased PD risk in *GBA1* mutation carriers have not been fully clarified. A dual interplay has been proposed for GCase and α-synuclein. On the one hand, GCase loss-of-function would lead to aberrant lysosomal protein degradation and neurotoxicity, whereas on the other hand α-synuclein may inhibit the activity of normal GCase (32). Accordingly, in postmortem samples of PD patients without *GBA1* mutations, a reduced GCase activity has been reported (33). The scenario is even more complex, considering that significant loss of GCase activity can cause neurodegeneration even in the absence of α-synuclein (34). To better elucidate the precise mechanisms linking *GBA1* with parkinsonism, a number of disease models have been developed, including both animal models and cell-based models. *GBA1* knockout mice and transgenic mouse lines carrying *GBA1* point mutations well recapitulate GD phenotype, with accumulation of α-synuclein and ubiquitinated proteins, the presence of typical “Gaucher cells” and inflammation (28), but they also prove to be appropriate for the study of the effects of GCase reduction in PD pathogenesis. In the knockout mice, for instance, the lysosomal defect demonstrated in neurons and astrocytes lacking *GBA1* was correlated with dysfunctional and fragmented mitochondria, pointing out a possible relationship between decreased GCase activity and impaired mitophagy, due to the inhibition of the degradation of mitochondria by the autophagy–lysosome pathway (35). However, it is important to note that *GBA1* mutations do not act only through defective lysosome pathway: on the contrary, GCase loss-of-function may be associated with multiple other pathogenic cellular mechanisms, including ER stress, calcium metabolism dysregulation, and neuroinflammation (36), that, altogether, might contribute to the aggregation of misfolded α-synuclein. In this context, any disease-modifying therapy designed to increase GCase levels would act at different pathogenic levels, with the final target to slow down the progressive aggregation of α-synuclein (37, 38). In view of the close linkage between GCase function and α-synuclein deposition, it could be questioned whether this lysosomal enzyme deficiency can also affect synaptic function. A relatively recent experimental study gives interesting insights about this issue: in a murine model of PD, where a subchronic conduritol-β-epoxide exposure induced GCase inhibition, Ginns and colleagues identified an early synaptic impairment, in the form of a reduction of striatal evoked dopamine release and altered synaptic plasticity markers, including post-synaptic density size and miRNA expression levels, together with glial activation within nigrostriatal pathway and abnormal α-synuclein accumulation (39). The effects of *GBA1* insufficiency on dopaminergic neurotransmission and synaptic function documented in this animal model fit with clinical observations. Indeed, patients carrying *GBA1* mutations show early striatal presynaptic dopaminergic dysfunction even before the onset of motor symptoms (40, 41). Further studies of individuals carrying a mutant *GBA1* allele, together with the development and characterization of different *GBA1* models, will help to clarify the mechanisms underlying the Parkinson’s–GD connection and to provide novel insights into the influence of diminished GCase activity on synaptic transmission, which could lead to develop novel therapeutic interventions.

## ER Stress

The ER represents a quality control system to check the correct protein folding, while misfolded or unfolded proteins are directed toward cytosol for the degradation by ER-associated degradation system (42). The accumulation of misfolded proteins inside the ER lumen, defined ER stress, is a toxic process to which the cell reacts by activating the unfolded protein response (UPR) (43), with the aim to restore ER homeostasis. Conversely, if the adaptive response is insufficient, the cell undergoes apoptosis. The involvement of ER stress has been demonstrated in several neurodegenerative conditions, including PD (44), and many reports prove the role of some PD-related genes in this cellular process. For example, differentiated PC12 cells with expression of A53T mutant α-synuclein show decreased proteasome activity and increased ER stress (45). Moreover, a recent study performed on iPSC-derived DAns carrying GBA-N370S mutation demonstrates the activation of UPR with upregulation of ER-resident chaperones (46). Given the existence of an interplay between mitochondrial and ER stress (47), also mitochondrial proteins such as Parkin and PTEN-induced putative kinase 1 (PINK1) have a crucial role in this mechanism. Activation of ER stress, mediated by mitofusin bridges occurring between defective mitochondria and the ER, has been reported in Drosophila PINK1 and Parkin mutants (48). Despite the growing interest in this field, the contribution of ER stress to neuronal death still needs further investigation, in view of a potential application in PD therapeutics (49). In this regard, interesting inputs come from a very recent study performed on three different rodent models of PD, in which the authors described an activation of RNA-like ER kinase (PERK) signaling, as well as in postmortem brain tissue derived from parkinsonian patients (50). PERK is a crucial ER stress sensor, whose chronic signaling blocks the translation of essential synaptic proteins, impacting neuronal survival and synaptic function. In these experimental settings, PERK inhibition exerted a neuroprotective effect, as evidenced by an increase in dopamine levels and in the expression of synaptic proteins. This once again highlights the relevance of synaptopathy in multiple aberrant mechanisms in PD.

## Mitochondrial Impairment

Most neurodegenerative diseases share a mitochondrial impairment as a major pathophysiological hallmark. Mitochondria are the main source of chemical energy for the cell and, as a consequence, their dysfunction leads to decreased levels of ATP and production of reactive oxygen species, which negatively impact neuronal physiology and, ultimately, cell survival (49). An impaired mitochondrial complex I activity has been demonstrated in the SNpc of PD patients (51). It is well established that many environmental toxins act as potent mitochondrial complex I inhibitors, and accordingly, different toxin-based experimental models of PD have been developed in order to reproduce mitochondrial dysfunction. MPTP, acting through its metabolite MPP^+^, causes loss of dopaminergic SNpc neurons, inducing parkinsonian features in mice and non-human primates (52). The same toxin has been used to develop MPTP-treated models with only partial dopaminergic deafferentation, in which an early reduction in spine density in both the caudate nucleus and putamen could be detected, as an early pathological hallmark of the disease (53). Rotenone, a largely used pesticide, is another mitochondrial complex I toxin, and its administration reproduces many histochemical and behavioral features of human PD in rodents and non-human primates, including selective nigrostriatal dopaminergic lesions and α-synuclein-positive cytoplasmic aggregates in nigral neurons (54). Such experimental evidence has been further confirmed by a number of epidemiological studies (55). Mitochondrial dysfunction and oxidative stress are tightly connected to PINK1, a serine/threonine kinase located in the intermembrane mitochondrial space and involved in the mitophagic pathway (56). *PINK1* loss-of-function mutations are linked to inherited early-onset forms of PD (57). In a mouse model carrying heterozygous PINK1 mutations, we identified early rearrangement within corticostriatal circuitry, expressed by selective impairment of LTP with a physiologically expressed LTD, in the absence of motor phenotype and dopaminergic neuronal loss (58). These observations were in line with the results of neuroimaging and physiological studies performed on *PINK1* heterozygous mutation carriers manifesting initial alterations in the nigrostriatal circuit (59, 60). Accordingly, the heterozygous condition related to familial parkinsonism represents an ideal preclinical model, which allows us to study early alterations occurring before the onset of motor signs, in a time window suitable to test potential novel disease modifying therapy (61). A number of attempts have been made to recreate the gene–environment interaction that might underlie disease pathogenesis. Recently, we exposed *PINK1* heterozygous knockout mice to rotenone, which was chronically administered at very low doses. Of interest, combination of gene mutation with minimal rotenone exposure was able to cause severe alterations of corticostriatal synaptic plasticity, to an extent similar to that observed in the *PINK1* homozygous knockout model (62, 63). The experimental use of toxins inducing mitochondrial impairment has contributed over the past decades to improve our knowledge on the pathogenic mechanisms of neurodegenerative diseases, in an attempt to develop neuroprotective agents and etiologic treatments.

## Conclusion and Future Directions

Over the past decades, major advances have been made in the understanding of the mechanisms involved in PD pathogenesis and the mechanisms that, furthermore, share common elements and contribute synergistically to neuronal dysfunction. Growing evidence attributes an undisputed central role to α-synuclein aggregation, also in view of its interaction with multiple processes, including intracellular trafficking, mitochondrial dysfunction, ER stress, and lysosomal dysfunction. However, there remain many unclear issues. The captivating prion-like hypothesis, according to which aggregated α-synuclein is trans-synaptically spread through the brain connectome, is still debated, as it does not fully recapitulate PD pathogenesis (64). Indeed, the pattern of spreading of Lewy-body pathology does not precisely match Braak’s theory, according to a number of studies examining postmortem PD samples (65). Yet, at cellular level, the pattern of distribution of α-synuclein aggregates appears to spare brainstem GABAergic neurons (66). Such evidence highlights some limitations to the “prion-like” theory and supports the need for a more comprehensive hypothesis that could take into consideration the selective neuronal susceptibility (64). In this complex scenario, many experimental models point toward the synapse as the primary site of PD pathology and considering synapse failure as a putative common denominator. Indeed, synaptopathy is an early event in PD pathogenesis in most phenotypic and genetic models reported so far (Table 1). Impairment of synaptic activity and plasticity at corticostriatal synapses represents a peculiar endophenotype, in distinct models of human movement disorders (67–69). In addition, these alterations have been shown to parallel time-dependent progression of cellular demise, thereby mimicking a very important disease stage, where potential disease-modifying treatments could be tested. Understanding the molecular events leading to synaptic dysfunction, achieved by the use of suitable PD animal models, will encourage the development of potential synapse-target therapies, in the hope of actively intervene on one of the mechanisms leading to PD pathogenesis.

**Table 1:** Synaptopathy in different animal models of Parkinson’s disease.

Animal model	Model generation	Motor behavior	Nigral dopaminergic neuron loss	Synaptic alterations	Reference
α-syn (1-120) transgenic mice	Expression of truncated human α-syn (1-120)	Reduced locomotion	NO	Age-dependent reduction in dopamine release	(12)

BAC transgenic mice (SNCA-OVX)	Overexpression of human wild-type α-syn	Normal (3 mo of age) Motor deficits (18 mo of age)	No (3 mo of age) Yes (18 mo of age)	Reduced firing rate of SNpc dopamine neurons (18 mo of age) Increased clustering of vesicles in dopamine terminals Deficit in dopamine release	(13)

Unilateral 6-OHDA rat model	Partial dopamine denervation	Mild motor alterations	Partial	Selective impairment of corticostriatal LTP with sparing of LTD	(14)

8- to 9-month-old *LRRK2* transgenic mice	Expression of G2019S mutant *LRRK2*	Hypoactivity	NO	Reduced firing rate of SNpc dopamine neurons Impaired evoked dopamine release Impairment of corticostriatal LTD	(15)

*LRRK2* BAC transgenic rats	Expression of G2019S or R1441C mutant *LRRK2*	L-DOPA-responsive motor dysfunction	NO	Reduced burst firing of SNpc dopamine neurons (R1441C rats) Impaired dopamine release	(18)

CBE mouse model	Subchronic CBE exposure to inhibit GCase	Motor impairments	Glial activation in nigrostriatal pathway	Reduced evoked striatal dopamine release Altered synaptic plasticity markers	(39)

Unilateral 6-OHDA mouse model	Dopamine denervation Evidence of activation of PERK signaling	Motor impairments Attenuation of motor deficits after PERK inhibition	YES Reduced neuron loss after PERK inhibition	Lower levels of striatal dopamine, with complete recovery after PERK inhibition Reduced expression of synaptic proteins (VAMP2 and SNAP25), partially reverted after PERK inhibition	(50)

*PINK1*^+/−^ mice	Heterozygous *PINK1* knockout mice	Normal	NO	Lower striatal dopamine release Selective impairment of corticostriatal LTP with sparing of LTD	(58)

*The table summarizes early synaptic impairments reported in different PD models*.

*α-syn, α-synuclein; BAC, bacterial artificial chromosome; SNpc, substantia nigra pars compacta; LTD, long-term depression; LTP, long-term potentiation; CBE, conduritol-β-epoxide; GCase, glucocerebrosidase; mo, months*.

## Author Contributions

PI and AP designed the study and wrote the paper. TS and MM prepared illustrations and revised the text. NM revised the text.

## Conflict of Interest Statement

The authors declare that the research was conducted in the absence of any commercial or financial relationships that could be construed as a potential conflict of interest.
